# Parental Stress in a Pediatric Ophthalmology Population

**DOI:** 10.3390/vision7040069

**Published:** 2023-10-26

**Authors:** Sachin Kalarn, Clare DeLaurentis, Zaid Bilgrami, Ryan Thompson, Osamah Saeedi, Janet Alexander, Mary Louise Collins, Allison Jensen, Le Tran Notarfrancesco, Moran Levin

**Affiliations:** 1 Department of Ophthalmology and Visual Sciences, University of Maryland School of Medicine, 419 West Redwood St., Suite 479, Baltimore, MD 21201, USAzaid.bilgrami@som.umaryland.edu (Z.B.); ryan.thompson@som.umaryland.edu (R.T.); osaeedi@som.umaryland.edu (O.S.); jalexander@som.umaryland.edu (J.A.); 2 Department of Ophthalmology, Greater Baltimore Medical Center, 6569 Charles St. #505, West Pavilion, Towson, MD 21204, USAajensen@gbmc.org (A.J.); 3 Department of Psychiatry, Kaiser Permanente, 4700 Sunset Boulevard, Los Angeles, CA 90025, USA; letnotar@gmail.com

**Keywords:** parental stress, stress, pediatric ophthalmology, ophthalmology

## Abstract

To determine the rate of parental stress within a pediatric ophthalmology population, parents in an urban or suburban community pediatric ophthalmology clinic were administered the Parental Stress Index Short Form survey. Demographic information and parental depression or anxiety data were collected and analyzed using an independent sample *t*-test and chi-squared analysis. Stress measures were recorded as percentiles. One hundred and twenty-one surveys revealed the following mean percentiles: Total Stress, 45.9 ± 22.4; Parental Distress (PD), 49.7 ± 19.8; and Parent Child Dysfunctional Interaction (P-CDI), 45.1 ± 23.6. The PD percentiles of the non-married parents, those with positive parental depression or anxiety scores, and those with a high school diploma or less were 55.9 ± 18.5 versus 45.2 ± 19.6, *p* < 0.01; 55.2 ± 18.6 versus 46.7 ± 19.9, *p* < 0.05; and 56.8 ± 18.2 versus 47.0 ± 19.8, *p* < 0.01, respectively. The parents with a high school diploma or less in a suburban environment demonstrated higher PD/P-CDI scores versus those of an urban population. Those with median household incomes (MHI) below USD 60,000 in both the total and suburban populations showed higher PD scores. There is no significant difference in parental stress between the pediatric ophthalmology patients and the general population. The parents who are unmarried, depressed, have a high school degree or less, or an MHI below USD 60,000 experience significantly higher stress levels.

## 1. Introduction

Parental stress is a growing concern and relevant consideration in the management of childhood diseases, with noteworthy implications in ophthalmologic care. This study aimed to determine the rate of parental stress within a pediatric ophthalmology population as well as identify the factors associated with stress. Higher stress levels have been found in the parents of children with several chronic illnesses, including but not limited to congenital heart disease, autism spectrum disorder, cancer, juvenile arthritis, diabetes, and cystic fibrosis [1,2,3]. These increased stress levels have been found to significantly impact the child–parent relationship, worsen the patients’ outcomes, and inhibit compliance with the treatment of childhood illnesses [1,4,5]. Further, increased stress levels are known to predispose people toward other comorbidities and diseases, including but not limited to anxiety and depression [6]. The stress of the caregiver is thought to be associated with certain coping mechanisms that could be unhelpful or even harmful to both the caregiver and the care recipient. Such responses to parental stress include, but are not limited to, an increasingly sedentary lifestyle, poor sleep, the increased use of tobacco products, and decreased compliance with medical care [7]. These family caregivers of visually impaired people have been described as “increasingly expected to function competently as informal extensions of healthcare systems… without formal training or preparation” in assisting their visually impaired family member with the activities of daily living and providing emotional support. Most often, these family caregivers (in the pediatric population, this is frequently the parent) have little support, without any form of compensation or financial reinforcement, which can influence the caregiver to abandon their own wellness [8]. One study identified a sample of family caregivers of visually impaired children and found 35.4% to be at risk of depression [8]. It is quite realistic to imagine the implications within the pediatric ophthalmology population as well as within the field of general ophthalmology. For example, the presence of blindness at birth or a young age (affecting 500,000 children per year), among other visual deficits, presents an unanticipated challenge to parents who may have expected to rear a child in excellent health [7]. It is logical to assume that the consequences of parental stress might be particularly demonstrated among the parents of children with ophthalmologic conditions. 

Ophthalmologic conditions present their own unique challenges and demands in terms of management, and, as in other fields of medicine or general life, parents must play an active role in their child’s care. The caregivers of children with ophthalmologic conditions are often responsible for administering eye drops, ensuring the wearing of glasses, and participating in the process of patching a child’s non-amblyopic eye, which are notably extremely difficult tasks, often resulting in limited success. For some diseases, such as amblyopia, treatment is only effective during the critical period of visual development, and insufficient treatment during that time may result in a permanent reduction in visual acuity [9]. Therefore, the parents’ participation and compliance with interventions are of the utmost importance, and thus, their health literacy, comfort, and confidence in providing excellent care are paramount. Parental stress has been reported to contribute to declines in developmental and adaptive functioning in children; one study in particular noted this for retinoblastoma [10]. Similarly, one study revealed that the parents in the highest stress level sample, as compared to the parents in the lowest stress level sample, had been shown to spend less time (up to nearly a 75% reduction) performing prescribed eye occlusion therapy following the infants’ cataract surgery [11]. 

Despite growing interest in identifying and measuring parental stress within pediatric disease populations, little is known about the various risk factors that contribute to parental stress in the pediatric ophthalmology population. Since limited healthcare already presents a significant obstacle to wellness in the American population, with recent statistics revealing that 10% of U.S. citizens lack complete health insurance, any and all other obstacles to care, such as parental stress levels, deserve sufficient attention and intervention [12]. It has been shown that having a lower socioeconomic status is associated with decreased maternal responsiveness [13]. Other studies have demonstrated associations between increased stress and single motherhood, as well as associations between increased stress and lower levels of education [14]. It must be considered that urban and suburban populations could contain different levels of stress at the baseline, which could be still different in rural populations or in different cities. While various studies point to the risk factors associated with parental stress, these risk factors have yet to be fully characterized. One currently ongoing systematic literature review is imploring for additional research on parental stress within ophthalmology for the following specific reasons: the dramatic incidence and prevalence of blindness and visual deficits in children globally, the known relationship between stress and other comorbidities, and the potential implications of parental stress as a preventable, though sometimes irreversible, obstacle to visual development and ophthalmologic prevention and treatment [7]. Most of the current research has focused on the effects of these interventions on the child or the family in general [15]. The goal of the Baltimore Pediatric Ophthalmology Parental Stress Index (B-POPSI) dataset is to investigate the potential effects of parental demographics on parental stress levels in a pediatric ophthalmology population in an urban and suburban population compared to the general population.

## 2. Materials and Methods

The target population for this study included parents in Maryland seeking tertiary-level pediatric ophthalmologic care for their children aged 0 to 12 years. Samples targeted in this study presented to the urban clinic at the University of Maryland Medical Center Downtown Campus and the suburban clinic at the Greater Baltimore Medical Center (GBMC) pediatric ophthalmology practice. Individual participants were selected from a sampling frame at the point of care in the clinical setting. Participants were administered a demographic survey and the Parental Stress Index Short Form (PSI-4 SF). If more than one parent was present at the encounter, the person who scheduled the appointment was asked to fill out the survey. A nonprobability sampling design was used such that all consecutive potential parents could be invited to participate. The goal was to invite as many parents to participate as possible, but given the limitations of time and personnel, this resulted in a convenience and purposive sample between May 2015 and May 2018. Survey distribution occurred on random days and times for four different providers (two at each location), and was dependent upon the availability of the research coordinators and medical student volunteers who distributed the surveys. Participation was possible when both the parent and research coordinator or medical student had sufficient time to discuss participation. Surveys were self-administered after a study representative described the purpose of the survey and inspected for completeness. Participants were informed that the study was intended to evaluate parental stress, and then consented for participation. Demographic data including parent and child gender, age, parental-reported ethnicity, marital status, highest education level, zip code, whether the patient was new to the clinic or established, and if there was self-reported history of parental depression or anxiety were collected via a parent-administered survey. Clinical ophthalmologic diagnosis, visual acuity, cycloplegic and manifest refraction, duration of disease, and historic information regarding treatments including patching, glasses, eye drops, and ocular procedures and surgery were also collected. Parents were excluded if they were younger than 18 years of age. 

This study adhered to the tenets of the Declaration of Helsinki, was approved by the Institutional Review Board at the University of Maryland and Greater Baltimore Medical Center, and conformed to the requirements of the United States Health Insurance Portability and Privacy Act. 

At the particular pediatric ophthalmology practice at the tertiary care center involved in this study, there are many patients who are referred to rule out ocular conditions associated with genetic syndromes, or those who are unable to participate in visual screening by their primary pediatrician due to functional impairments. As a result, this study site experiences a higher prevalence of patients with pre-existing developmental disorders when compared to the general population (40% versus 17%) [16]. There is also substantial evidence of increased risk of parental stress in parents of children with neurodevelopmental delays and genetic syndromes [17,18,19]. The decision was made to exclude children with certain known genetic syndromes or developmental delay to minimize confounding variables and optimize generalizability. (Children with such conditions are disproportionally referred for screening to this study’s practice and are known to have higher stress levels in parents.) The PSI-4 SF is a validated 36-question survey that produces scores for parental stress across four subscales: Parental Distress (PD), Parent–child Dysfunctional Interaction (P-CDI), Difficult Child (DC), and Total Stress. PD measures the level of distress a parent is experiencing in their role as a parent due to personal factors. P-CDI measures a parent’s perception that the child does not meet their expectations or that their interactions with the child are not reinforcing the parental role. DC measures a child’s basic behavioral characteristics that affect how easy or difficult the child is to manage. Finally, Total Score measures the overall stress a parent is feeling within the role of a parent, and is an additive measure of the other three sub-scores. Each of the 36 items is graded on a five-point Likert scale, from 1 (strongly disagree) to 5 (strongly agree). PSI scores less than the 85th percentile are considered within the normal range, scores between the 85th and 90th percentile are considered high, and scores above the 90th percentile are viewed as clinically significant for parental stress. Percentile threshold scores are generated from population normative data developed for the survey [20]. The primary outcome measure is the percentile of parental stress compared to the general population. The secondary outcome measure is the percentile of parental stress scores in urban versus suburban populations. A Defensive Response (DR) sub-score provides an assessment of which respondents approach the PSI-4 SF with a strong bias. Respondents with a high DR sub-score try to present themselves with the most favorable impression or to minimize their stress or problems. Median household income (MHI) data for parent-provided zip codes were obtained from an online, searchable U.S. Census Bureau database, and were calculated with respect to the year that the survey was completed [21]. Reporting of census data was based on city or census designated place (CPD), and zip codes may include multiple cities. To account for this, MHI was averaged over all cities in a particular zip code. Based on this income data, parents were grouped into MHI greater than $60,000 or less than $60,000; $60,000 was the calculated living wage for Baltimore county based on the Massachusetts Institute of Technology Living Wage Calculator at this time [22]. The surveys were analyzed using SPSS software (IBM, Armonk, NY, USA, version 26). 

An independent samples t-test was performed on all dichotomous demographic variable and primary outcome scores (PSI-4 SF sub-scores) to identify high-risk factors. A chi-squared test was used to determine demographic differences between the two survey locations. Statistical significance was considered to be a *p*-value of <0.05. The effects of individual ophthalmologic diagnosis and medical and surgical interventions on parental stress were not determined as part of this study, and will be investigated in future work.

## 3. Results

Demographics: A total of 121 parents were included in this study with a predominate population of mothers (84.3%) that was similar between the two sites (*p* > 0.05). As aforementioned, respondents with a high DR sub-score may to present themselves with a more favorable impression or minimize their stress or problems; thus, 37 surveys with a high DR sub-score were removed from analysis to provide an unbiased parental stress assessment [20]. The population was composed of a Caucasian (47.9%) population, an African American (41.3%) population, and the remaining population, Hispanic or other. A large proportion of the Caucasian subgroup came from the suburban clinic location (77.6%), whereas the urban clinic location population was composed mainly of African Americans (88.0%). Most of the population was married (57.9%). The majority were also from the suburban location (87.7% versus 31.3%, *p* < 0.01). A minority of the population had a highest education level of a high-school graduate degree or less (28.1%) with a significant proportion originating from the urban clinic location (39.1% versus 5.3%, *p* < 0.01). Some 35.5% of parents reported having depression or anxiety, with no significant difference between the two sites. There was a similar proportion of girls to boys in the overall patient population, without any significant difference between the two sites (Table 1). 

**Figure 1:**
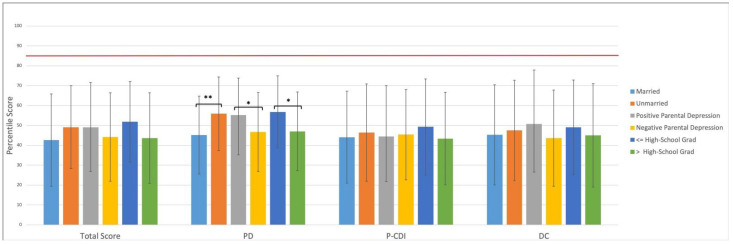
Graphic representation of percentile scores for the entire studied patient population (*n* = 121) broken down by Total and Sub-scores, and by whether parents were married versus unmarried, whether parents reported parental depression, and whether the patient was male versus female. Red bar represents the clinically significant stress percentile threshold. * denotes a *p* < 0.05; ** denotes a *p* < 0.01.

**Figure 2:**
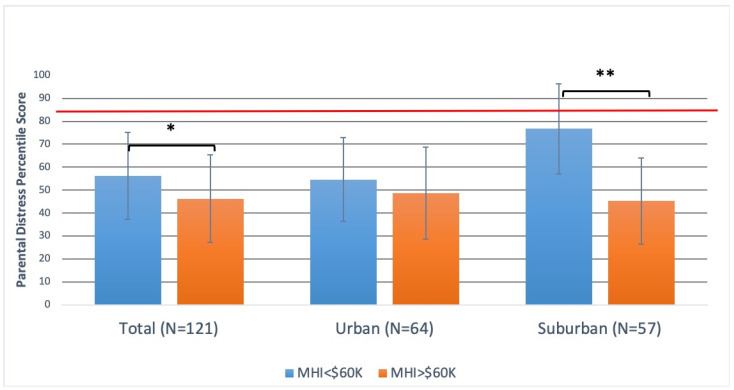
Parental Distress percentile scores for parents who have a median household income ($) greater than or less than $60,000. Total population and subgroups at the urban and suburban locations are represented. Red bar represents the clinically significant stress percentile threshold. * denotes a *p* < 0.05; ** denotes a *p* < 0.01.

Average MHI was $66,905.68 ± 23,848.58, with a significantly higher MHI in the suburban population compared with University of Maryland Medical Center Downtown Campus ($81,460.96 ± 18,767.79 versus $53,757.08 ± 20,076.99, *p* < 0.01). A majority of the 33.9% of the study population who had an MHI less than $60,000 presented to the urban clinic, representing 59.4% of participants, compared to 5.3% in the suburban clinic (*p* < 0.01). PSI-4 SF Percentiles: The overall population percentile score for Total Stress was 45.9 ± 22.4, Parental Distress was 49.7 ± 22.6, Parent–Child Dysfunctional Interaction was 45.1 ± 23.6, and Difficult Child was 46.2 ± 25.4. Based on the 85th-percentile threshold for significant stress defined by the PSI-4 SF survey, no parent met this criterion in the studied populations. Total stress and all sub-score percentiles were similar between the urban and suburban locations (*p* > 0.05). The Parental Distress percentile score in this population was significantly higher for parents who were unmarried (55.9 ± 18.5 versus 45.2 ± 19.6, *p* < 0.01), reported a history of parental anxiety or depression (55.2 ± 18.6 versus 46.7 ± 19.9, *p* < 0.05), and had a highest education level of a high-school graduate degree or less (56.8 ± 18.2 versus 47.0 ± 19.8, *p* < 0.01). There were no significant differences in Total Score, P-CDI, or DC and those parents who were unmarried, reported positive parental depression, those with a high-school graduate degree or less, or whether they were new versus established patients (Figure 1). A subgroup analysis of urban population showed no differences in Total Score percentile or any subgroup percentile scores. The suburban population demonstrated no significant difference in the child’s gender, or in parents who were unmarried or reported positive parental anxiety or depression. The suburban subgroup demonstrated significantly higher PD percentile scores for parents who had an MHI of less than $60,000 (76.7 ± 19.7 versus 45.2 ± 18.7, *p* < 0.01) (Figure 2). A similar comparison amongst the urban population was not found to be significant. There was also a significantly negative yet weak correlation, upon bivariate linear regression, between MHI and PD percentile scores (β = −0.183, R2 = 0.034, *p* <0.05). Although not included as part of the analysis, parents who had children with a developmental delay or known genetic disorder (*n* = 11) demonstrated significantly higher stress percentiles for Total and all sub-scores (Total stress: 61.5 ± 27.2 versus 38.0 ± 24.9, *p* < 0.01; PD: 63.7 ± 29.3 versus 40.4 ± 24.8, *p* < 0.01; P-CDI: 58.2 ± 28.1 versus 39.6 ± 23.9, *p* < 0.05; DC: 60.6 ± 28.6 versus 40.2 ± 26.0, *p* < 0.05).

## 4. Discussion

Overall, the pediatric ophthalmology population identified in this study did not display a significant difference in parental stress, including sub-score analysis compared with the general population. This study does, however, identify certain specific risk factors for parental stress. There existed significantly higher Parental Distress levels in parents who are unmarried, have a high school diploma or less education, and those who had self-reported depression or anxiety. Similar findings have been reported in the literature, and similar risk factors have also been associated more directly with visual difficulty. One analysis of United States adults identified lower income and lower educational level, in addition to other factors (including race and insurance), as being associated with increased barriers to care and visual difficulty [23]. Another revealed that patients with limited education (high school diploma or less) expressed that they were “unaware of need” and were thus associated with fewer/less frequent ophthalmic health screenings (among other obstacles). Those with a referral from their primary care provider were associated with increased ophthalmologic preventative care [24]. Among parents of children with retinoblastoma, higher levels of parental stress are reported in parents previously diagnosed with depression and those with developmentally delayed children [25]. Another study among parents of children with retinoblastoma found that parents who were married reported lower stress levels than those who were unmarried [10]. This study on parental stress within pediatric ophthalmology did find a significantly negative, though, weak, correlation between median household income and parental stress, with lower household income being associated with higher levels of parental stress. This finding is consistent with previous studies that show a negative correlation with material responsiveness to their children and poverty [13]. These results also suggest Parental Distress to be significantly higher in the total population as well as in subgroup analysis within suburban parents who had an MHI of less than $60,000. Interestingly, this suggests that lower income in the context of a suburban, community-based clinic contributes to higher stress levels as compared to an urban, significantly lower average MHI, which did not demonstrate a similar difference.

Identification of these parental populations within the field of pediatric ophthalmology that have displayed increased levels of stress in the greater Baltimore population, including parents who have a history of experiencing depression, those parents who are single/unmarried, those whose highest level of education meets a high school degree or less, as well as those with a median household income of $60,000 or less, is helpful for the clinician to bear in mind, as special attention may be considered for additional resources for these families. For example, it may yield benefit to provide additional time, clearer education, additional care supplies, transportation, emotional reassurance and support, and any other sources of positive feedback for healthy, sustainable, habitual caregiving to be dedicated for qualifying patients in clinic to mitigate stress and improve outcomes. As expressed earlier in the Vision Detroit Project, support from the primary care provider was associated with increased ability to obtain eye exams [24]. In fact, The Vision Detroit Project also identified a successful 5-Point Teaching Intervention in an urban community that both increased health literacy and increased follow-up vision screening in populations with similar risk factors including education limited to a high school level or less [26]. 

Limitations to this study on parental stress within a pediatric ophthalmology population do exist. To begin, the small sample size does not allow for full appreciation of the weight of varying factors. Further, though both an urban population within Baltimore and a suburban population in the greater Baltimore area were intentionally analyzed to provide a more generalizable study population, there still persists limited generalizability as the study population did not represent any pediatric ophthalmology clinics in the rural setting. Additional studies assessing parental stress within pediatric ophthalmology in rural settings are indicated, as well as studies in the urban and suburban communities of other large cities. A non-response bias may be present within this study, as data regarding the number of parents who refused to participate are not available for analysis. Further, the nature of this study being based on surveys allows for the possibility of response bias, though such possibilities were minimized. The PSI-4 SF provides a Defensive Response sub-score that screens for parents who approach the survey with a potentially significant bias. By removing surveys that reached a pre-defined threshold, this data set provides an assessment of parental stress within a pediatric ophthalmology population with a more limited bias. Weaknesses in this study could further include variable administration protocols, where surveys were completed either at the beginning or end of a patient visit, potentially introducing response bias. There was no significant difference uncovered between established and new patients. However, since new patients were not asked if they had prior pediatric ophthalmology care, patients may have been incorrectly categorized, resulting in an underestimation of a true difference. Additionally, average MHI may be inaccurate for some zip codes that encompass multiple cities with varying MHI. This is a limitation in the categorization of data from the US Census Bureau. One additional potential weakness in this study could include a confounding bias contributed by race in the suburban versus urban distinction. 

These factors should be stratified in future research. Lastly, no significant difference was determined between sex of the parent in this population, of which the majority were mothers. However, it remains unknown whether the presenting parent was the identified primary caregiver. It also remains unknown if that parent may be receiving assistance that could affect stress responses. Such questions could be added to surveys in future work for consideration. It would be beneficial to investigate the spectrum of ophthalmic pathology and the associated levels and quality of parental stress in the future. Thus, future work aims to utilize the data collected in the B-POPSI dataset to determine the effect of various pediatric ophthalmology diagnoses and treatments on parental stress, and to determine the effects of high parental stress on treatment compliance. Based on these findings, pediatric ophthalmologists can consider targeting specific populations at risk for high parental stress to offer additional support and interventions that will improve patient care and lead to improved compliance. Similar studies conducted at varying institutions may help local practices identify unique stresses and at-risk populations. Further studies are also essential to identify successful strategies to address these risk factors associated with parental stress in order to optimize care for the pediatric ophthalmology patient.

## 5. Conclusions

The results of this study suggest that parents of patients within the field of pediatric ophthalmology do not exhibit significantly higher stress levels than the general population. However, certain qualifiers are suggested by this study to correlate with higher levels of stress. Parents who are unmarried, have a history of anxiety or depression, have a high school graduate degree or less, or have a median household income of less than $60,000 in particular displayed significantly higher parental stress levels, and will likely benefit from additional resources, attention, and support in the clinical and home setting. Future research can aim to investigate other plausible factors associated with parental stress, including different types and severity levels along the full spectrum of ophthalmic pathology, the sex of primary caregiver, and the presence of assistance. Future research can include larger sample sizes and more types of populations.

## Figures and Tables

**Table 1:** Demographic data for both of the two study locations: urban (University of Maryland) and suburban (Greater Baltimore Medical Center).

	Total	Urban	Suburban	
n (%)	121	64 (52.9%)	57 (47.1%)	
Parent–Female: *n* (%)	102 (84.3%)	55 (85.9%)	47 (82.5%)	*p* > 0.05
Parent Age (years)	37.36 ± 7.37	34.89 ± 7.35	40.12 ± 6.21	*p* < 0.01
AA/Black	50	44	6	*p* < 0.05
Caucasian	58	13	45	*p* < 0.05
Hispanic	1	1	0	
Other	12	6	6	
Married *n* (%)	70 (57.9%)	20 (31.3%)	50 (87.7%)	*p* < 0.01
HS Grad and Less	34 (28.1%)	31 (39.1%)	3 (5.3%)	*p* < 0.01
Parental Depression	43 (35.5%)	28 (48.4%)	18 (31.6%)	*p* > 0.05
Patient–Female: *n* (%)	62 (51.2%)	37 (57.8%)	34 (59.6%)	*p* > 0.05
Patient Age (Months)	73.31 ± 49.02	63.97 ± 49.77	85.49 ± 43.76	*p* < 0.05
AA/Black	50	44	6	*p* < 0.05
Caucasian	54	11	43	*p* < 0.05
Hispanic	1	1	0	
Other	16	8	8	
Median Household Income (MHI)	$66,904.68 ± 23,848.48	$53,757.08 ± 20,076.99	$81,460.96 ± 18,767.79	*p* < 0.01
MHI ≤ $60 K	41 (33.9%)	38 (59.4%)	3 (5.3%)	*p* < 0.01
Total Score	45.9 ± 22.4	44.6 ± 22.6	47.5 ± 22.1	*p* > 0.05
PD	49.7 ± 19.8	52.8 ± 19.0	46.3 ± 20.2	*p* > 0.05
P-CDI	45.1 ± 23.6	42.7 ± 25.1	47.7 ± 21.8	*p* > 0.05
DC	46.2 ± 25.4	42.3 ± 25.7	50.5 ± 24.5	*p* > 0.05
Established Patient	87 (71.9%)	37 (57.8%)	50 (87.7%)	*p* < 0.01

Abbreviations: PD—Parental Distress, P-CDI—Parent–Child Dysfunctional Interaction, DC—Difficult Child.

## Data Availability

Publicly available datasets were analyzed in this study. This data can be found here: https://livingwage.mit.edu/counties/24005; https://www.census.gov/quickfacts/MD; https://www.cdc.gov/nchs/data/nhsr/nhsr177.pdf (accessed on 16 February 2021). Other datasets analyzed including the Parenting Stress Index™, Fourth Edition.
